# The complete chloroplast genome of a Korean endemic plant *Chrysosplenium aureobracteatum* Y.I. Kim & Y.D. Kim (Saxifragaceae)

**DOI:** 10.1080/23802359.2018.1450668

**Published:** 2018-03-16

**Authors:** Yong-In Kim, Jung-Hoon Lee, Young-Dong Kim

**Affiliations:** a International Biological Material Research Center, Korea Research Institute of Bioscience and Biotechnology, Daejeon, Korea;; b Department of Life Science, Hallym University, Chuncheon, Korea

**Keywords:** Saxifragaceae, *Chrysosplenium*, chloroplast genome, endemic species

## Abstract

*Chrysosplenium aureobracteatum* is endemic to Korea. This species is primarily found in Gyeonggi Province and Gangwon Province in the central part of the Korean Peninsula. The complete chloroplast genome (cpDNA) sequence of *C. aureobracteatum* was characterized using Illumina paired-end sequencing. The cpDNA is 153,102 bp in length and contains a pair of 26,017-bp inverted repeats (IRs), a large single copy (LSC) region of 83,752 bp, and a small single copy (SSC) region of 17,316 bp. It contains 131 genes, including 84 protein-coding genes (78 PCG species), eight ribosomal RNA genes (four RNA species), 37 transfer RNA genes (30 tRNA species), and one pseudogene (accD). The overall G + C content of the whole genome is 37.3%. Phylogenetic analysis based on 24 chloroplast genomes indicates that *C. aureobracteatum* is closely related to *Bergenia scopulosa.*

*Chrysosplenium aureobracteatum* Y.I. Kim & Y.D. Kim is a recently discovered perennial herbaceous plant belonging to Saxifragaceae and is an endemic species of Korea. This species is primarily found in Gyeonggi and Gangwon Province in the central part of the Korean Peninsula. They grow in moist, shaded valleys and mountain slopes approximately 600–900 m above sea level (Kim and Kim 2015). In this study, we report and characterize the plastid genome of *C. aureobracteatum*. These data will provide important information for the development of a protection strategy for the species and the study of genome diversity.

The plant material was sampled at Mt. Gwangduk (Gangwon Province, South Korea), the sampling site for the type specimens. The voucher specimens (KYI-2009032) were deposited in the herbarium of Hallym University (HHU). The whole-genomic DNA data were sequenced using the Illumina Hiseq 2500 platform. The paired-end data set comprised 45,500,518 reads (length 2 × 101 bp). Paired reads were trimmed using gBBDuK (Bushnell 2016). The trimmed paired-read set was assembled using the Geneious *de novo* assembler. *De novo* assembly generated 224,333 contigs. The largest contig was 128,344 bp with an average coverage depth of 6594.2 (minimum coverage 20). Gene annotations of chloroplast genome sequence of *C. aureobracteatum* were performed by using Geneious v 8.1.9 (Biomatters Ltd., Auckland, New Zealand) via comparison with chloroplast genome of *Bergenia scopulosa* (KY412195). The annotated chloroplast genome sequence was submitted to the GenBank under the accession number of MG878089.

The complete chloroplast genome of *C. aureobracteatum* has a total length of 153,102 bp, with a pair of inverted repeats (IRs) of 26,017 bp that separate a large single copy (LSC) region of 83,752 bp and a small single copy (SSC) region of 17,316 bp (Figure 1). The chloroplast genome of *C. aureobracteatum* contained 131 genes, including 84 protein-coding genes, 37 tRNA genes, and eight ribosomal RNA genes. Among these genes, 14 genes (atpF, ndhA, ndhB, petB, petD, rpl16, rpoC1, rps16, trnA-UGC, trnG-GCC, trnI-GAU, trnK-UUU, trnL-UAA, and trnV-UAC) have one intron, and three genes (clpP, rps12, ycf3) have two introns. One gene (accD) were inferred to be pseudogene. Most of the genes occurred as a single copy. However, six protein-coding genes (ndhB, rpl2, rpl23, rps7, rps12, and ycf2), seven tRNA genes (trnA-UGC, trnI-CAU, trnI-GAU, trnL-CAA, trnN-GUU, trnR-ACG, and trnV-GAC), and four rRNA genes (rrn16, rrn23, rrn4.5, and rrn5) in the IR regions were duplicated. The overall G + C content of *C. aureobracteatum* chloroplast genome is 37.3%, respectively.

**Figure 1.**
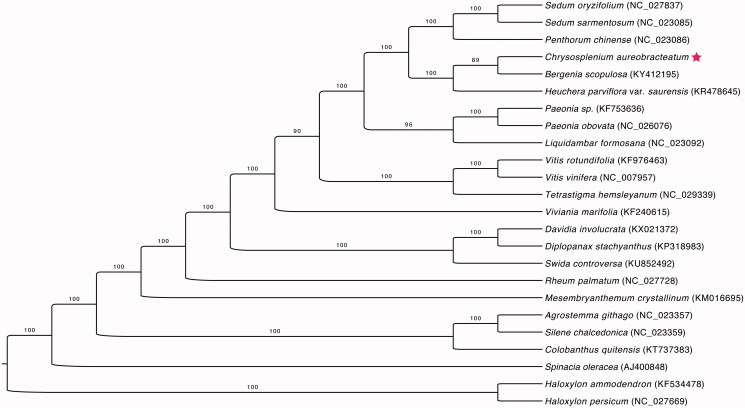
Maximum likelihood phylogenetic tree based on 23 complete chloroplast genomes. The number on each node indicates the bootstrap value. Solid star marks the newly sequenced *C. aureobracteatum* in this study.

Phylogenetic analysis was performed using chloroplast coding gene sequences of *C. aureobracteatum* and those of 23 related species of Saxifragales, including two *haloxylon* species as outgroups. These chloroplast genomes were aligned using MAFFT (Katoh and Standley 2013). A maximum likelihood tree was generated using raxmlGUI v. 1.31 (Silvestro and Michalak 2012) based on a GTR + G model with ML + rapid bootstrap 1000 replicates. As shown in the highly resolved ML phylogenetic tree (Figure 1), all of the species in the order Saxifragales formed a clade, and *C. aureobracteatum* was most closely related to *B. scopulosa*.
